# Reproductive Characteristics of Cockatiels (*Nymphicus hollandicus*) Maintained in Captivityand Receiving Madagascar Cockroach (*Gromphadorhina portentosa*) Meal

**DOI:** 10.3390/ani9060312

**Published:** 2019-05-31

**Authors:** Thatijanne Santos Gonzaga de Carvalho, Carlos Eduardo do Prado Saad, Marcelo Esposito, Peter Bitencourt Faria, Renata Ribeiro Alvarenga, Livia Geraldi Ferreira, Walter Motta Ferreira, Tarcisio Moraes Gonçalves, Marcio Gilberto Zangeronimo

**Affiliations:** 1 Departament of Animal Science, Federal University of Lavras, Lavras, Minas Gerais 37200-000, Brazil,; thatijanne@yahoo.com.br (T.S.G.d.C.); saadzoo@ufla.br (C.E.d.P.S.); marceloesposit@gmail.com (M.E.); renata.alvarenga@ufla.br (R.R.A.); tarcisio@ufla.br (T.M.G.); 2 Department of Veterinary Medicine, Federal University of Lavras, Lavras, Minas Gerais 37200-000, Brazil; peter@ufla.br (P.B.F.); liviagferreira@gmail.com (L.G.F.); 3 Veterinary School, Federal University of Minas Gerais, Belo Horizonte, Minas Gerais 31270-901, Brazil; waltermf@ufmg.br

**Keywords:** cockatoo, psittacid, nutrition, reproduction, Madagascar cockroach

## Abstract

**Simple Summary:**

This research article describes the effects of including a Madagascar cockroach meal in the feed of cockatiels on their reproductive characteristics. The cockatiel is one of the most common species of the *Psittaciformes* order adopted as a pet and, despite this, information on the reproductive characteristics of this species and how nutrition can influence these characteristics is still scarce. However, the evaluation of this ingredient in the feed of cockatiels has not yet been performed. We observed that the inclusion of a cockroach meal in the feeds of cockatiels increased the number of viable chicks with 1 day of life, decreased the number of days for new laying, increased egg width and shape index, and influenced its lipid composition. Therefore, the present study provides important information about an alternative protein ingredient that can assist researchers and breeders in the management of these birds, ensuring better health for the animals as well as gains in productivity.

**Abstract:**

The aim of this study was to evaluate the use of a Madagascar cockroach (*Gromphadorhina portentosa*) meal in the feed of cockatiels (*Nymphicus hollandicus*) in captivity and its influence on the reproductive characteristics of these birds. Twelve pairs of birds were used during two subsequent reproductive cycles of 130 days each, with time divided into four phases: laying, incubation, rearing of chicks until 30 days of age, and return to the new laying phase. The pairs were divided into two groups: a control group, which received a commercial diet for psittacines + a mixture of seeds, and a test group, which received the same diet as the control group except for the addition of a Madagascar cockroach meal in a ratio of 14 g of commercial food to 1 g of cockroach meal (6.6%). After hatching, chicks remained with their parents until 30 days of age. Subsequently, the chicks were transferred to another room and monitored until the 90th day of life. The inclusion of cockroach meal did not influence (*p* > 0.05) the intake of commercial food and mixture of seeds during the reproductive phases evaluated, except for feed intake, which was increased relative to control values (*p* = 0.02) in the return-to-laying phase. Yolk cholesterol content, egg width and egg shape index were increased with the inclusion of the cockroach meal, whereas the number of days to return to the new laying phase was reduced compared to the control (*p* = 0.02). The number of eggs laid decreased (*p* < 0.05) with the inclusion of the cockroach meal; however, the percentage of hatching was higher in the test group than in the control group (*p* < 0.05). No significant effect (*p* > 0.05) of dietary treatment was observed on the number of viable chicks at 1, 30 and 90 days of age or on the contents of most fatty acids present in the yolk. The findings of this study indicate that a Madagascar cockroach meal can be used as an alternative feedstuff in the diets for cockatiels and can lead to minor improvements in reproductive characteristics when replacing 6.6% of the commercial pelleted diet.

## 1. Introduction

Cockatiel (*Nymphicus hollandicus*) rearing has been increasing in popularity, mainly due to the color diversity of these birds, their capacity to learn and imitate sounds, and their small size, docile nature, and ease of handling [1,2]. After the budgerigar (*Melopsittacus undulatus*), the cockatiel is the most common species of Psittaciformes adopted as a pet, and it is commonly found in breeding places and commercial establishments [3,4].

Cockatiels are classified as granivorous, and their natural diet is composed of seeds, fruits, leaves, flowers [5], and small insects—especially during the reproductive season [4,6]. However, when these birds are maintained in captivity, seed-based diets associated with commercial pelleted psittacine pellets are commonly provided because of their widespread availability, practicality and low cost. Nevertheless, unconventional foods, such as insect meal, are used in some countries and have been tested in other animals such as broilers [7], fish [8] and rats [9] with positive results. However, there is a lack of information on the influence of nutrition on cockatiel reproduction. The observation that cockatiels in the wild consume insects during the reproductive season suggests that this type of food might influence the reproductive success of the species.

In general, insects in animal feed are good sources of protein and fat. They are easy to breed and grow well on dried and cooked waste materials [10]. For these reasons, they are produced industrially as animal feeds to reduce the use of conventional feedstuffs such as corn and soybean meal in commercial diets. However, as different species and ages (e.g., larvae vs. adults) of insects can be used, the nutritional values of diets containing insects may vary significantly [11]. Reports in literature have shown that crude protein of different insects (*Tenebrio molitor*, *Hermetia illucens*, *Gromphadorhina portentosa*, *Gryllus assimilis*, *Musca domestica*, *Nauphoeta cinerea* and *Zophobas morio*) can range from 36% to 70%, lipids from 13% to 34% and gross energy from 5063 to 7332 Kcal/kg [10,12,13]. In broilers [14,15], pigs [16] and fishes [12], studies have shown that the protein from insects is similar to the protein from other, conventional sources, such as fishmeal and soybean meal. For cockatiels, there is no scientific information available to evaluate the safety of the inclusion of this type of food in the diet. In addition to the wide variation of the chemical composition of the insects, the low levels of calcium in insects might compromise not only eggshell quality but also chick growth. The inclusion of this kind of food in the diet of reproductive cockatiels warrants evaluation since energy imbalances, the presence or absence of essential fatty acids, and low calcium levels can directly influence the health of these animals [5,17].

The Madagascar cockroach (*Gromphadorhina portentosa*) is a tropical cockroach that has excellent conditions for survival at 23 °C to 26 °C and around 30% humidity. Under these circumstances, the nymphs can mature in 3 months, and the adults can live for 2 or 3 years having 2 or 3 broods of 20 to 50 live young a year [18]. The chemical composition of a Madagascar cockroach meal varies around 67% of crude protein, 17% of total fat, 5063 kcal/kg of gross energy and 0.25% of calcium with calcium: phosphorus ratio of 0.27 [12,19]. There are no reports of the use of the cockroach meal in cockatiel diets. We hypothesized that the inclusion of the insect meal in the cockatiel diet does not influence the reproductive performance of these birds. The overall aim of this study was to evaluate the influence of the use of the Madagascar cockroach (*G. portentosa*) meal as a dietary supplement on the reproduction of cockatiels (*N. hollandicus*) maintained in captivity.

## 2. Materials and Methods

### 2.1. Location, Animals and Experimental Design

The research was carried out in the Wild Animals Sector of the Animal Science Department at the Federal University of Lavras, in Lavras-MG, Brazil, from October 2016 to March 2017. The experimental protocol was approved by the Ethics Committee in Animal use of the same institution under the number 080/16.

Twelve pairs of cockatiels (*N. hollandicus*), with an average age of 4.2 ± 0.3 years and a body weight of 100.1 ± 9.5 g and 107.3 ± 9.1 g for males and females, respectively, were used. The birds were housed under both natural and artificial light in a room with an area of approximately 20 m^2^, a window and an exhaust fan. Individual pairs of cockatiels were randomly placed in galvanized wire cages (80 cm wide, 45 cm deep and 40 cm high) with three wooden perches. Three round ceramic bowls of 10 cm diameter each were placed in each cage, one for the seed mixture, one for the pelleted diet (CC Parrots Complete Food for Birds, BioTron, Rio Claro, São Paulo, Brazil) and one for water. In addition, each cage contained a nest box for breeding (33 cm wide, 16.5 cm deep and 15 cm high) constructed of medium-density fiber (MDF), with wood shavings in its interior. All animals were clinically healthy (determined via physical examination) and fit for reproduction and had been paired in their existing pairs since 8 months of age. The pairs were evaluated during two subsequent reproductive cycles. The environment lighting provided artificial light via fluorescent, white, tubular lamps (1.2 m long, 120 V, 32 W, Ourolux, São Paulo, Brazil) located approximately 2 m from the cages. The lighting system was connected to a timer (TE 30 Elcon, Belo Horizonte, Minas Gerais, Brazil) to supply 15 h of artificial light per day [20]. The experimental period was 130 days. The mean temperature and humidity of the room during the experimental period were 24.4 ± 1.7 °C and 67.0 ± 5.8%.

The birds were evaluated in five different phases:

(a) Laying: The laying phase from the appearance of the first egg to the last egg of each pair before incubation.
(b) Incubation: The phase of the last egg laying until the hatching of the first chick of each pair.
(c) Post hatching: The phase from the hatching of the first chick of each pair until the moment when the last chick completed 30 days of life.
(d) Return to reproduction: The phase from the exit of the last chick from the nest box until the appearance of the first egg of each pair.
(e) After leaving the nest boxes, chicks continued to be evaluated until 90 days of age, when the number and final weight of the birds were recorded.

### 2.2. Experimental Procedure

At the beginning of the experiment, the pairs were divided into two groups: A control group (receiving seed mixture + pelleted commercial food) and a treatment group (seed mixture + pelleted commercial food mixed with cockroach meal, with 1 g of commercial cockroach meal added per 14 g of pelleted food, i.e., 6.6% of the diet). The number of viable chicks produced in the reproductive cycle prior to the experiment was used to allocate the pairs into groups. A randomized complete block design was used. The cockroach meal was composed of crushed adult insects (Madagascar cockroach, *G. portentosa*, Vida Proteina Indústria e Comércio LTDA, Neropolis-GO, Brazil).

The seed mixture consisted of 50% millet, 30% canary seed, 15% oats and 5% sunflower seed; this mixture is commonly used in cockatiel breeding facilities in Brazil [21]. The pelleted diet was specific for psittacines. Both the seed mixture and the pelleted commercial food with or without Madagascar cockroach meal were provided ad libitum. The chemical and energetic compositions of the diets provided during the experimental period were analyzed at the Animal Research Laboratory of the Animal Science Department at the Federal University of Lavras and are presented in Table 1.

The feed intake of each pair was measured daily by providing a known amount of food. Leftovers and wastes were measured the following day. Water was supplied ad libitum. Environmental temperature (°C) and relative humidity (%) were measured daily by a digital thermohygrometer (Model 7666.02.0.00, Incoterm, Porto Alegre, RS, Brazil).

Pairs underwent a 15-day acclimation period to the experimental diets prior to the nest placement in the cages. The nest boxes were inspected once a day at 7:00 a.m. for egg laying. On the day of laying, the eggs were weighed on a precision scale (Coleman 0.1 g - BN1200, Viçosa, MG, Brazil), and handled with latex gloves. Each egg was labeled with the cage number and laying order by using a permanent black marker (Model 1.0 mm, Pilot BT, São Paulo, Brazil). Height and width were then measured using a pachymeter (Model Ws8 Dc-6, 150 mm, Western, São Paulo, Brazil). The eggs remained inside the nest box for a maximum period of 28 days, at which point they were considered unhatched eggs. Cracked, dirty and broken eggs were counted and discarded. Mean incubation time and the percentages of laid and hatched eggs were recorded.

After hatching, the chicks remained with their parents for 30 days and were then transferred to other cages (33 cm wide, 16.5 cm deep and 15 cm high) and fed the same diet they had received in their parents’ cage. The chicks were observed until the 90th day of life to determine viability in this period. The number of chicks per cage ranged from one to three.

The feed intake of each pair and the number of days until their return to reproductive activity were evaluated after the chicks were removed from the cages. After their return to reproductive activity, the first three eggs of each pair were collected, packed in round plastic bowls with a diameter of 5 cm and stored in a refrigerator (Consul Bem Estar 405 Litros, São Paulo, Brazil) at 10 °C for a maximum period of 15 days from the date of laying of the first egg of each pair. At the time of egg removal from the nest box, egg height and width (mm) were measured. In addition, the weight (g) before and after storage was recorded.

Measurement of the specific gravity of the eggs was performed after storage [22]. Subsequently, the eggs were cut in half with thin-tipped stainless steel scissors (Metzembaum Scissors 12 cm curve, Golgran, São Caetano do Sul, São Paulo, Brazil) and transferred to a flat surface (glass plate). The yolk and albumen were separated manually. Yolk and albumen heights were measured with a digital pachymeter. Yolks were weighed and had their color evaluated by means of subjective comparison with a colorimetric fan (DSM Yolk Fan TM, Amsterdam, The Netherlands). The eggshells were washed, dried at room temperature for 48 h, weighed on an analytical balance, and evaluated for thickness by using a digital pachymeter at two points in the center-transverse area of the eggshell. Albumen weight (g), shape index (ratio between the smallest and largest diameter of the egg), Haugh unit (UH = 100 log (H + 7.57 − 1.7 W^0.37^), where H is the albumen height in millimeters and W is the egg mass in grams) [23], calcium percentage of the eggshell, and protein percentage were also measured. For the analysis of cholesterol and fatty acids, lipids were extracted according to the procedures described by Folch et al. [24] after being esterified and separated [25]. Cholesterol was quantified by colorimetry [26]. Fatty acid analysis was performed by gas chromatography on a Shimadzu GC 2010 gas chromatograph (Shimadzu Corporation, Kiyoto, Japan) [27]. The analyses were performed at the Laboratory of Animal Products Inspection of the Department of Veterinary Medicine at the Federal University of Lavras.

### 2.3. Statistical Analysis

The data were evaluated for normality by the Shapiro-Wilk test, for homoscedasticity by the Breusch-Pagan test, and for independence of the errors by the Durbin-Watson test. Variables that did not meet the analysis of variance assumptions were analyzed via nonparametric analysis, and the differences between means were evaluated by the Wilcoxon score obtained from the NPAR1WAY procedure of SAS^®^ [28]. Data that did meet the ANOVA assumptions were then submitted to covariance analysis, including the number of chicks obtained in the previous reproductive cycle as a covariate. This analysis was performed using the PROC GLM procedure, and repeated measures analysis was performed by using the PROC MIXED procedure. In the mixed procedure, variance and covariance were based on the smaller value of the Akaike Information Criterion. The averages were estimated by least square means, and the differences were evaluated via the Tukey-Kramer test.

## 3. Results

The dietary treatment did not influence (*p* > 0.05) the consumption of seeds and commercial food by the pairs in any of the experimental phases evaluated (Table 2). Relative to the control diet, the inclusion of cockroach meal decreased (*p* < 0.05) the number of eggs laid but increased (*p* < 0.05) the percentage of hatching. There were no differences between treatments (*p* > 0.05) in the number of viable chicks at 1, 30 and 90 days of age. However, in the test group, the number of days until new laying was decreased (*p* < 0.05) and feed intake after chick exit was increased relative to the control values. The inclusion of cockroach meal did not influence (*p* > 0.05) the consumption of protein and lipids in any of the evaluated phases.

Regarding the egg characteristics, greater width, higher shape index and lower yolk pigmentation were observed (*p* < 0.05) in the group of birds that received the cockroach meal (Table 3) than in the control group. Regarding the lipid profile, higher levels of capric acid, margaric acid, and cholesterol were observed (*p* < 0.05) with the use of cockroach meal than with the control diet (Table 4). There were no differences in the other physicochemical egg characteristics.

## 4. Discussion

In the present study, the inclusion of Madagascar cockroach meal in the cockatiel diet improved some reproductive parameters, including the number of days to return to egg laying, eggshell resistance and egg cholesterol level. Cockroach meal reduced the number of eggs laid but did not influence the number of chicks per couple due to the increase in hatching rate. There are no reports in the literature regarding the use of insect meal in cockatiel diets; the present study is the first to evaluate the influence of this ingredient on the reproductive characteristics of cockatiels.

The use of alternative protein sources, especially insect meal, can impair the laying performance of birds [29]. This is due to the amino acid imbalance and also to the presence of antinutritional factors in feedstuffs. In the present study, a low level of cockroach meal (1 g for each 14 g of commercial diet, i.e., 6.6% of the total diet) was selected to avoid possible decreases in reproductive performance due to nutritional imbalances [30]. Studies determining the protein and energy requirements for psittacines are scarce. There is evidence that cockatiels can tolerate diets with high levels of protein [31]. In the present study, the crude protein content of the commercial food was 17.1%, whereas that of the diet supplemented with Madagascar cockroach meal was 18.8%. As the consumption of this food is relatively low when compared to the seed mixture, the increase in protein and lipid intake was not significant.

The use of insect meal in animal diets is not new [9]. However, most research on this topic is recent [8,29,32] and has focused on fish and amphibians [33]. Furthermore, studies with Madagascar cockroach (*G. portentosa*) are scarce in the literature. Most are with black soldier fly (*H. illucens*), housefly (*M. domestica*), and yellow mealworm (*T. molitor*). Despite the limited number of studies, the use of insects as an alternative ingredient in animal diets has been mentioned by the United Nations’ Food and Agriculture Organization [34]. Insects have an adequate nutritional composition for inclusion in the diet of some species [35] and have high nutritional value in terms of proteins, fats, minerals and vitamins [36,37]. Some studies have investigated the use of insect meals in poultry feeding and have demonstrated that the protein from this ingredient is similar to that from other, conventional sources [29,38].

In livestock, for example, replacing up to 15% of soybean meal with mealworms in broiler diets improve body weight and feed intake, but negatively affect feed efficiency and intestinal morphology [39]. The authors concluded that low levels of this feedstuff may be more suitable. In another study, maggot meal replaced fish meal when this ingredient was added in 10% of the total diet [13]. However, in this case, higher maggot inclusion negatively affected chicken growth and performance, and additional methionine was needed in the broiler diet. Already Schiavone et al. [40] have shown that the compositional data of partially defatted and a highly defatted black soldier fly larvae meal are good sources of dietary protein. In this study, both meals showed a higher crude protein content than soybean meal. For laying hens, when maggot meal was fed to replace fish meal, no significant impact on performance had been observed, but egg yolk cholesterol and calcium concentration were significantly reduced [41]. *H. illucens* larvae meal also showed to be a suitable alternative protein source for laying hens even if the complete replacement of soybean meal [29]. Nevertheless, these authors concluded that further investigation is necessary to avoid the negative effects on feed intake.

In the present study, most of the reproductive parameters did not differ between the diets. The inclusion of cockroach meal increased the shape index from 74% to 80%. Shape index values between 72% and 76% are considered as standard for laying hens and indicate that the eggs are resistant to breakage [42]. The finding that the addition of cockroach meal increased the egg shape index may be related to the lower number of eggs laid by birds receiving this supplement, which may have favored the allocation of nutrients to these eggs. In addition, the increase in the yolk cholesterol content in the treatment group relative to the control group may have been related to the higher number of viable chicks obtained with the cockroach meal. Cholesterol is important for embryo and posthatching development since embryos and chicks have no enzymes for the synthesis of this compound in the first days of life [43].

The cholesterol concentration of egg yolk may be influenced by diet composition [44]. Hossain and Blair [45] observed lower serum cholesterol and triglycerides in broilers when chitin was used at 50 g/kg of inclusion in diets. According to Prajapati and Patel [46], the chitin present in insect meals is be able to attract bile acids and free fatty acids during the digestion process. In fact, it is known that the chitin of insects is difficult to digest by domestic poultry [47,48]. However, these results were not observed in the present study. Considering that the seed intake is the main source of nutrients for cockatiels, it can be stated that the amount of chitin ingested was relatively low when compared to poultry production. The slight increase in the cholesterol content in the eggs may be due to the small increase in the lipid content of the birds fed diets containing the insect meal. As cholesterol is a precursor of sexual hormones, probably this could contribute to influencing the reproductive performance in insect-fed birds [49]. Bird species listed in increasing concentrations of cholesterol in yolk are chicken, pheasant, quail, turkey, duck, goose, and dove, with a total range of 12.77 mg to 21.99 mg of cholesterol per gram of yolk [34]. In this study, mean cholesterol concentration was 5.22 mg/g yolk for the control group and 6.00 mg/g yolk for the pairs that received the cockroach meal. These values differ from the cholesterol contents reported for quails (17.94 mg/g yolk) [50] and laying hens (16.08 mg/g yolk) [51].

Yolk color can vary from pale yellow to dark orange depending on the amount of carotenoids present in the diet [52]. Wild insects have been found to contain a spectrum of carotenoids, including β-carotene, lutein and zeaxanthin [19,53]. Reports in the literature have shown that *G. portentosa*, in its adult form, contains approximately 66.3 mg/kg (in DM basis) of carotene, equivalent to 386 IU/kg of vitamin A [19]. According to manufacturer information, the commercial food used in the present study contain minimum of 8330 IU/kg of vitamin A. No reports were found in the literature demonstrating the capacity of psittacine species to convert carotenoids into vitamin A. Thus, it is believed that the lower intake of carotene from the diet containing the insect meal may have reduced the deposition of this nutrient in the egg. Even so, the low yolk pigmentation in the treatment group in this study did not negatively influence the number of viable chicks after hatching. According to Koutsos et al. [5], cockatiels can be maintained for over a year on carotenoid-free diets with no loss of feather coloration. Furthermore, chicks hatched from these birds develop normal yellow and orange feather colors even when fed carotenoid-free diets.

In general, bird reproduction entails high nutritional costs, particularly during laying [54]. However, in the current study, there was only a tendency (*p* = 0.07) of higher feed intake in this phase. Oonincx and Dierenfeld [19] have shown that *G. portentosa*, in its adult form, contain higher levels of lipid, protein, iron, zinc, copper and lower levels of calcium and manganese when compared to commercial food used in the present study. According to Veloso et al. [55], crude protein level has little influence on feed intake by birds; the quality and balance of amino acids and other nutrients are more important. In this research, there were no significant differences in commercial diet and seed consumption between the groups in most of the experimental periods evaluated. Feed intake was stimulated using cockroach meal only in the period after the exit of chicks from the nest box.

In nature, some psittacines reproduce at the time of increased availability of protein-rich foods. In these species, the intake of amino acids may be a great determinant of reproductive efficiency, as is the case for *N. hollandicus* [31]. In addition, laying rate is positively correlated with the protein content of the diet [56,57,58]. Therefore, insect consumption may provide the additional protein necessary to maintain reproductive activity at certain times of the year [59]. In captivity, the supply of protein in adequate quantity and of adequate quality is important throughout the reproductive phase. In the present study, although egg production was reduced with the inclusion of cockroach meal, this addition improved the reproductive performance of cockatiels; it decreased the number of days to return to the next laying phase and improved the physical and nutritional characteristics of the eggs.

Changes in the general composition of the diet can affect the composition of yolk lipids [60]. Studies have shown that daily energy intake, whether above or below requirements, affects lipid deposition in the yolk but has little or no effect on the lipid composition of yolk [61,62]. In the present study, the crude energy content of the commercial diet was 5153 kcal/kg, whereas the one supplemented with cockroach meal was 5246 kcal/kg. According to Koutsos et al. [5], cockatiels require 3497 kcal/kg of metabolizable energy for maintenance. Some studies have reported the metabolizable energy values of some insects evaluated in broiler chickens. For example, metabolizable energy values of 4275 kcal/kg [63] for housefly larvae (*M. domestica*), 4027 kcal/kg for Tenebrio (*T. molitor*) and 4151 kcal/kg for the black soldier fly (*H. illucens*) have been reported [10]. These observations suggest that for *N. hollandicus*, the energetic levels of the food supplemented with cockroach meal was sufficient for maintenance.

In altricial birds such as cockatiels, the survival of chicks after hatching depends on their parents’ behavior and the composition of their diet [64]. In the present study, the viability and survival of chicks at 30 and 90 days were not influenced by the use of cockroach meal, suggesting that this ingredient can be safely used in the diet of cockatiels of reproductive age.

In general, the inclusion of cockroach meal in cockatiel diets improved the reproductive characteristics of birds. However, studies that consider higher levels of cockroach meal than were used in the present study should be conducted.

## 5. Conclusions

Cockroach (*G. portentosa*) meal can be used as an alternative source of protein in the diets of cockatiels (*N. hollandicus*). This meal improved, little expressively, the reproductive characteristics of birds when added at 6.6% of the commercial pelleted diet.

## Figures and Tables

**Table 1:** Chemical composition of the diets of cockatiels (*N. hollandicus*) maintained in captivity.

Component	Seed Mixture ^a^	Commercial Food ^b^ (CF)	Cockroach Meal ^c^ (CM)	CF + CM
Dry matter (%)	88.47	89.08	93.62	89.62
Crude protein (%)	14.23	17.08	57.79	18.81
Energy (kcal/kg)	3904	5153	5784	5246
Ether extract (%)	8.33	8.84	22.05	9.32
Mineral matter (%)	4.00	4.28	3.56	4.98
Calcium (%) ^e^	0.41	2.50	0.48	2.38
Phosphorus (%) ^e^	0.65	0.55	1.76	0.65

^a^ Seed mixture consisted of 50% millet, 30% canary seed, 15% oats and 5% sunflower seed. ^b^ CC Parrots Complete Food for Birds; BioTron, Rio Claro, São Paulo, Brazil. ^c^ Madagascar cockroach meal (*G. portentosa*); Vida Proteína Indústria e Comércio LTDA. ^d^ Commercial food (14 g) + Madagascar cockroach meal (1 g); 6.6% inclusion. ^e^ Maximum values guaranteed by the manufacturers.

**Table 2:** Feed intake and reproductive characteristics of cockatiels (*N. hollandicus*) maintained in captivity and receiving diets with or without Madagascar cockroach (*G. portentosa*) meal.

Variable	Control	Cockroach Meal *	*p* =	SEM
Seed intake (g/day)				
Laying phase	15.66	16.05	0.71	1.03
Incubation phase	18.38	17.56	0.21	0.61
Post-hatching phase	24.32	24.70	0.81	2.74
Return to reproduction phase	16.01	13.86	0.65	6.13
Feed Intake (g/day)				
Laying phase	1.55	2.73	0.07	0.57
Incubation phase	2.20	2.94	0.29	0.65
Post-hatching phase	8.99	11.54	0.42	5.33
Return to reproduction phase	2.30	3.78	0.02	1.21
Crude Protein Intake (g/day)				
Laying phase	2.49	2.80	0.10	0.11
Incubation phase	2.99	3.05	0.19	0.12
Post-hatching phase	5.00	5.69	0.58	0.44
Return to reproduction phase	2.67	2.68	0.86	0.11
Fat Intake (g/day)				
Laying phase	1.44	1.59	0.14	0.06
Incubation phase	1.73	1.74	0.86	0.07
Post-hatching phase	2.82	3.13	0.68	0.24
Return to reproduction phase	1.54	1.51	0.62	0.06
Number of eggs laid	4.33	3.53	0.02	
% of hatching	51.3	56.7	0.04	
Mean period of incubation (days)	19	18	0.78	1.35
Number of Viable Chicks/pair				
1 day	2.33	2.00	0.46	
30 days	1.83	1.71	0.32	
90 days	1.33	1.29	0.56	
Number of days to return to reproduction	10.17	9.00	0.02	1.15

SEM: standard error of the mean. * Madagascar cockroach meal (1 g) added to commercial food (14 g) (6.6% inclusion).

**Table 3:** Egg characteristics of cockatiels (*N. hollandicus*) maintained in captivity and receiving diets with or without Madagascar cockroach (*G. portentosa*) meal.

Variable	Control	Cockroach Meal*	*p* =	SEM
Egg Characteristics in the Nest Box				
Weight (g)	5.38	5.61	0.32	0.51
Height (mm)	25.98	25.19	0.22	1.42
Width (mm)	19.30	20.12	0.03	0.81
Shape index (%)	0.74	0.80	0.01	0.05
Egg Characteristics After Storage				
Total weight (g)	5.35	5.56	0.37	0.53
Density (g/cm^3^)	1.04	1.03	0.31	0.01
Eggshell weight (g)	0.33	0.35	0.33	0.05
Eggshell thickness (mm)	0.22	0.20	0.30	0.05
Eggshell calcium (%)	29.10	30.80	0.42	3.50
Yolk weight (g)	1.34	1.37	0.61	0.14
Yolk height (mm)	8.96	8.78	0.47	0.55
Yolk color	8.07	6.87	0.04	1.26
Protein in yolk (%)	16.56	17.65	0.18	1.30
Albumen weight (g)	3.57	3.85	0.16	0.41
Albumen height (mm)	3.00	3.08	0.75	0.58
Protein in albumen (%)	9.59	10.14	0.36	0.99
Haugh unit	80.88	81.15	0.86	3.43

SEM: standard error of the mean. * Madagascar cockroach meal (1 g) added to commercial food (14 g) (6.6% inclusion).

**Table 4:** Lipid profile of egg yolk of cockatiels (*N. hollandicus*) maintained in captivity and receiving diets with or without Madagascar cockroach (*G. portentosa*) meal.

Variable	Control	Cockroach Meal*	*p* =	SEM
Cholesterol (mg/g of yolk)	5.22	6.00	0.04	49.38
Saturated Fatty Acids				
C10:0 capric	0.0051	0.0089	<0.01	<0.01
C12:0 lauric	0.0163	0.0178	0.64	<0.01
C14:0 myristic	0.4625	0.4872	0.62	0.08
C15:0 pentadecylic	0.0152	0.0180	0.22	<0.01
C16:0 palmitic	25.1575	25.5385	0.51	0.96
C17:0 margaric	0.0824	0.0928	0.03	<0.01
C18:0 stearic	8.2454	8.4259	0.69	0.76
C20:0 arachidonic	0.0325	0.0339	0.67	<0.01
C22:0 behenic	0.0074	0.0080	0.72	<0.01
Total	34.0243	34.6309	0.34	1.06
Monounsaturated Fatty Acids				
C14:1 myristoleic	0.0890	0.0829	0.74	0.03
C15:1 pentadecanoic	0.0141	0.0182	0.13	<0.01
C16:1 palmitoleic	4.4652	4.3227	0.86	1.36
C17:1 cis-10-heptadecanoic	0.0428	0.0515	0.08	<0.01
C18:1ω9T elaidic	0.1650	0.1765	0.31	0.02
C18:1ω9C oleic	43.6096	44.1952	0.64	2.09
C20:1 gadoleic	0.0977	0.1033	0.78	0.03
Total	48.4834	48.9504	0.75	2.45
Polyunsaturated Fatty Acids				
C18:2ω6 linoleic	12.0332	11.1941	0.41	1.70
C18:3ω6 γ-linolenic	0.2583	0.2151	0.28	0.07
C18:3ω3α-linolenic	0.1400	0.1272	0.52	0.03
C20:2 eicosadienoic	0.0597	0.0610	0.93	0.03
C20:3ω6 dihomo-gamma-linolenic	0.0945	0.0775	0.06	0.01
C20:4ω6 arachidonic	4.2526	4.0720	0.49	0.44
C20:5ω3 timnodonic	0.0365	0.0320	0.54	0.01
C22:6ω3 docosahexaenoic	0.6175	0.6398	0.72	0.10
Total	17.4494	16.3672	0.36	1.96
Totalunsaturated Fatty Acids	65.9328	65.3176	0.34	1.06
Total ω3	0.7939	0.7990	0.94	0.12
Total ω6	16.6386	15.5587	0.35	1.90
ω6/ω3	20.9523	19.9179	0.54	2.83
∆9-desaturase C16	14.9592	14.2870	0.75	3.55
∆9-desaturase C18	84.0820	83.9988	0.92	1.35
Elongase C16-C18	63.6109	63.8163	0.88	2.24
Thioesterase C16-14	98.1955	98.1246	0.72	0.33

SEM: standard error of the mean. * Madagascar cockroach meal (1 g) added to commercial food (14 g) (6.6% inclusion).
